# ProtVista: visualization of protein sequence annotations

**DOI:** 10.1093/bioinformatics/btx120

**Published:** 2017-03-07

**Authors:** Xavier Watkins, Leyla J. Garcia, Sangya Pundir, Maria J. Martin, the UniProt Consortium

**Affiliations:** 1 EMBL-EBI, Hinxton, UK; 2 Open Targets Wellcome Genome Campus, Hinxton, Cambridgeshire CB10 1SD, UK; 3 SIB Swiss Institute of Bioinformatics, Centre Medical Universitaire, Geneva 4, Switzerland; 4 Protein Information Resource, Georgetown University Medical Center, Washington, DC 20007, USA; 5 Protein Information Resource, University of Delaware, Newark, DE 19711, USA

## Abstract

**Summary:** ProtVista is a comprehensive visualization tool for the graphical representation of protein sequence features in the UniProt Knowledgebase, experimental proteomics and variation public datasets. The complexity and relationships in this wealth of data pose a challenge in interpretation. Integrative visualization approaches such as provided by ProtVista are thus essential for researchers to understand the data and, for instance, discover patterns affecting function and disease associations.

**Availability and Implementation:** ProtVista is a JavaScript component released as an open source project under the Apache 2 License. Documentation and source code are available at http://ebi-uniprot.github.io/ProtVista/.

**Contact:** martin@ebi.ac.uk

**Supplementary information:** Supplementary data are available at *Bioinformatics* online.

## 1 Introduction

With the continuous growth in biological data, integration and visualization are increasingly important to aid interpretation. UniProt (The UniProt Consortium, 2015) provides various protein sequence annotations, or ‘features’, such as domains, sites, post-translational modifications and variants from multiple sources. Visualizing these features together enables the identification of patterns that might affect protein function; for instance, deleterious variants co-localized with a known important protein motif or structural feature. While browsers and tools exist for genomic sequences and specific types of protein features such as structures, e.g. PDBe for secondary structure and InterPro for domains, there is currently no highly interactive tool which allows the visualization of a wide range of protein sequence features together in the same space.

ProtVista is implemented using JavaScript and makes extensive use of D3 (https://d3js.org/), a library for producing dynamic, interactive data visualizations in web browsers. Our viewer is implemented as a BioJS component (Corpas *et al.*, 2014) to ensure interoperability with other visualization tools, and its source code is publicly available on GitHub. It makes use of the Protein API (http://www.ebi.ac.uk/proteins/api/doc/swagger/), which provides the required data through different endpoints (features, variants and peptides) as XML and JSON. Each endpoint is called asynchronously thus reducing the waiting time for end-users. This implementation makes it easy to add more data endpoints from UniProt and/or other external data sources as they become available in the future.

## 2 Visualization

**Fig. 1.**
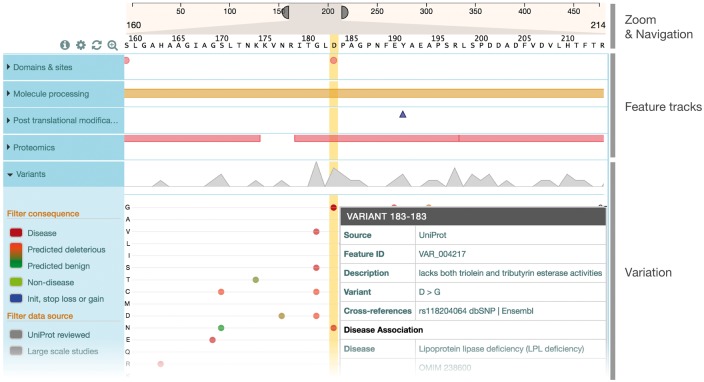
Simplified view of ProtVista for Human Lipoprotein lipase (P06858) To investigate the human Lipoprotein lipase enzyme’s involvement in the Lipoprotein lipase deficiency (LPL deficiency), we can look at the potential effect of variants on active sites. Clicking on the active site at position 183 highlights three disease variants in the same position. Clicking on these variants brings up a popup window containing more information about the variants, showing direct relation with the disease

ProtVista’s display consists of three sections (see Fig. 1). The first, located at the top, is used for navigation and zooming. It represents the full length of the protein sequence. Elements on both sides of the component can be dragged to specify the zoom level, as well as navigating along the sequence. The second section, on the left, consists of categories of the feature types. The third and main section of the visualization is the area where the features are displayed in tracks. Each category track is collapsed by default, providing a feature-dependant overview of all the features in the category. If multiple features are present at the same position, a bumping algorithm will vertically reduce the size of the features and arrange them to ensure they don’t overlap. When a category is expanded, this overview disappears and each type track then contains a representation of its own features in the same way as the overview does. In this area, it is possible to use the mouse scroll wheel as well as gestures to change the zoom level and navigate along the protein sequence. Clicking on a feature highlights the area covered by the feature, allowing for quick discovery of overlapping features, as well as highlighting the corresponding amino-acid sequence if the zoom level allows their display. A tooltip also appears and displays more information about the feature: exact position, description, scientific evidence and data source with cross-reference if available. The viewer was iteratively improved through a User Centered Design process (see Supplementary Information), including feedback from the scientific community from the early stages of development. A dictionary of shapes and colors to represent protein features was defined with input from various expert resources (InterPro, Pfam and Intact) to ensure consistency. The variation track provides a different way of displaying amino acid changes using a matrix-based approach to map the changes to their sequence position. The y-axis of the matrix represents all amino acids, grouped by chemical properties, as well as deletions and stop gained mutations while the x-axis is the position in the protein sequence. Compared to other paradigms such as sequence logos (Schneider and Stephens, 1990), this approach gives equal importance to each variant for a given position. A set of filters allows users to select variants by their consequence (disease association, predicted deleteriousness) or data source (large-scale studies or UniProtKB). Color categories allows users to quickly see the severity of the variant: burgundy (disease) and light green (benign) for UniProtKB reviewed, and a luminance scale based on the product of SIFT (Kumar *et al.*, 2009) and PolyPhen (Adzhubei *et al.*, 2010) predictions for large scale studies. Tooltips contain more information relevant to the disease association and provenance of the variant data.

## 3 Summary

ProtVista provides the scientific community with a visualization tool integrating information available about UniProtKB proteins from curated, automatic and imported sources. It also allows users to add custom data services using the features JSON format. ProtVista offers an intuitive and compact representation of protein features, making it easier to highlight different data relationships that might otherwise be unclear or difficult to grasp. It has been developed as a JavaScript component for easy integration within any website and is already used by the UniProt website (www.uniprot.org), the Open Targets platform (www.targetvalidation.org) and the EMBL-EBI Enzyme Portal (www.ebi.ac.uk/enzymeportal/; Alcántara *et al.*, 2013). As part of our future plans, we are exploring interactive integration with public web based genomic visualization tools and ways to upload user data directly to the visualization tool.

## Funding

This work has been supported by the National Institutes of Health (NIH), National Human Genome Research Institute (NHGRI) and National Institute of General Medical Sciences (NIGMS) grant U41HG007822; the European Molecular Biology Laboratory core funds; and Open Targets.

*Conflict of Interest*: none declared.

## Supplementary Material

Supplementary Data
