# Supplementary material for: ProtVista: visualization of protein sequence annotations
Source: Bioinformatics. 2017 Mar 7;33(13):2040–1. doi: 10.1093/bioinformatics/btx120 (PMC5963392; doi:10.1093/bioinformatics/btx120)
Supplement: Supplementary Data [file btx120_supp.doc]

*Supplementary material for* doi: 10.1093/bioinformatics/xxxxx

| Supplementary material for ProtVista: an interactive visualisation of protein sequence annotations Xavier Watkins^1,2^, Leyla J. Garcia^1^, Sangya Pundir^1^, Maria J. Martin^1^,* and the UniProt Consortium^1,3,4,5^.  ^1^EMBL-EBI and ^2^Open Targets, Wellcome Trust Genome Campus, Hinxton, Cambridgeshire, CB10 1SD, UK, ^2^SIB Swiss Institute of Bioinformatics, Centre Medical Universitaire, 1 rue Michel Servet, 1211 Geneva 4, Switzerland, ^3^ Protein Information Resource, Georgetown University Medical Center, 3300 Whitehaven Street North West, Suite 1200, Washington, DC 20007, USA and ^4^Protein Information Resource, University of Delaware, 15 Innovation Way, Suite 205, Newark, DE 19711, USA  *To whom correspondence should be addressed.  **Software Availability:**  **Contact:** martin@ebi.ac.uk |
| --- |

# USER AND DEVELOPER DOCUMENTATION

Available at <https://github.com/ebi-uniprot/ProtVista>

# USER EXPERIENCE REPORTS

We followed a User Centred Design (UCD) methodology to develop the ProtVista viewer. UCD advocates involving users in every stage of the design and development process. We began by researching requirements with users and investigating existing approaches in genome/ protein feature visualisations. We then moved to the ideation phase, where we created sketches and early design mockups based on our research and tested these with users. We provided users with tasks to perform using our prototypes and observed the positives and negatives in their experience. This approach enabled us to make changes while still in the early design phase and iterate the design using mockups. We then implemented the design and continued to validate interaction details with users through usability testing of live versions. Involving users in our design process also allowed us to better prioritise features based on user feedback. A useful reference for applying UCD for Bioinformatics services: <http://www.ebi.ac.uk/training/online/course/user-experience-design/> .

Here we report our User Experience testing information.

Table 2. User experience report

| **Testing round** | **Location** | **Number of participants** | **Types of institutes** | **Testing phase** | **Backgrounds** | **Usability takeaways** | **Resulting changes** |
| --- | --- | --- | --- | --- | --- | --- | --- |
| Nov-12 | London and Cambridge, UK | 9 | Academia | Conceptual paper prototypes | Proteomics, cancer research, heart disease research | Highlighting all features on a single sequence track too cluttered. Repeat of colours on different tracks is not confusing. 'Reading' the information track by track. Would want to select one feature at a time. | - Each category on a different track.  - Colour definition finalised.  - Selection behaviour changed. |
| Mar-15 | Washington DC, USA | 5 | Academia and industry | Interactive prototype | Biomedical research, genomics, metagenomics, disease genetics | Not clear how to scroll left and right once zoomed-in. Would like to be able to hide tracks. Would like to be able to zoom in at different levels. Don't always click on the feature after seeing tooltip. Variation view not always clear. | - Zoom and drag on mouse behaviour added.  - Settings icon added to hide/ show tracks.  - Tooltips removed to test if users will try clicking instead. |
| Apr-16 | Munich, Germany | 8 | Academia | Interactive prototype | Proteomics, Bioinformatics | Would like to be able to hide tracks. Zoom behaviour not fully obvious. Detailed feedback about content of tooltips. Don't always click on the feature after seeing tooltip. Would like to be able to highlight a feature's position. Variation view scalability issues. | - New variation matrix view designed.  - Handles added to ruler for zoom and drag.  - Clicking on a feature highlights position vertically. |

After releasing ProtVista, we continue to use UCD approaches to validate further changes and developments. For example, we were looking to iterate the colour encoding of the consequence of predicted variants as the first version did not work well for color blindness. We thought that using a transparency scale rather a colour scale would improve this issue. We created an A/B test for our new mockup and the old design in a short survey and sent this to potential users. The final design decision was supported by feedback from 61 users, including 10 colour-blind users.

# PERFORMANCE PROFILING

Here we present a performance profiling for three UniProtKB protein entries. The first one, A0A089H9A2, is a non-well characterized protein with just seven features, the second one, P05067, is a well-characterized proteins with 611 features, and the third one, Q8WZ42, is also a well-characterized protein with a very long sequence of about 35,000 amino acids and 16,444 features. Loading, scripting, rendering and painting times in milliseconds can be found in Table 2. Times were collected using the Timeline tab on Google Chrome Web Developers Tools, timing is defined as follows:

- Loading*:* parse HTML, send request and receive response.
- Scripting*:* script execution including animation, garbage collection, function call and event handling.
- Rendering*:* layout display, style recalculation and scrolling.
- Painting*:* painting, layers composition and image handling.

Table 2. Performance profiling

| **Protein**  **Task** | **Non-well characterized protein**  **A0A089H9A2** | **Well charaterized protein**  **P05067** | **Well characterized long protein**  **Q8WZ42** |
| --- | --- | --- | --- |
| ***Loading*** | 292.4 | 410.6 | 5582.2 |
| ***Scripting*** | 6.9 | 40.4 | 1933.8 |
| ***Rendering*** | 21.1 | 35.5 | 449.5 |
| ***Painting*** | 2.2 | 5.1 | 35.9 |
